# In-Vehicle Alcohol Detection Using Low-Cost Sensors and Genetic Algorithms to Aid in the Drinking and Driving Detection

**DOI:** 10.3390/s21227752

**Published:** 2021-11-21

**Authors:** Jose M. Celaya-Padilla, Jonathan S. Romero-González, Carlos E. Galvan-Tejada, Jorge I. Galvan-Tejada, Huizilopoztli Luna-García, Jose G. Arceo-Olague, Nadia K. Gamboa-Rosales, Claudia Sifuentes-Gallardo, Antonio Martinez-Torteya, José I. De la Rosa, Hamurabi Gamboa-Rosales

**Affiliations:** 1 Unidad Académica de Ingeniería Eléctrica, Universidad Autónoma de Zacatecas, Jardín Juárez 147, Centro, Zacatecas 98000, Mexico; jose.celaya@uaz.edu.mx (J.M.C.-P.); jona95rg@gmail.com (J.S.R.-G.); ericgalvan@uaz.edu.mx (C.E.G.-T.); gatejo@uaz.edu.mx (J.I.G.-T.); hlugar@uaz.edu.mx (H.L.-G.); arceojg@uaz.edu.mx (J.G.A.-O.); nkgamboarosales@uaz.edu.mx (N.K.G.-R.); clausifuen@uaz.edu.mx (C.S.-G.); vargasj@uaz.edu.mx (J.I.D.l.R.); 2 Cátedras-CONACyT, Consejo Nacional de Ciencia y Tecnología, Ciudad de México 03940, Mexico; 3 Escuela de Ingeniería y Tecnologías, Universidad de Monterrey, San Pedro Garza García 66238, Mexico; antonio.martinez@udem.edu

**Keywords:** drinking and driving, smart vehicle, smart infotainment, alcohol detection, genetic algorithm

## Abstract

Worldwide, motor vehicle accidents are one of the leading causes of death, with alcohol-related accidents playing a significant role, particularly in child death. Aiming to aid in the prevention of this type of accidents, a novel non-invasive method capable of detecting the presence of alcohol inside a motor vehicle is presented. The proposed methodology uses a series of low-cost alcohol MQ3 sensors located inside the vehicle, whose signals are stored, standardized, time-adjusted, and transformed into 5 s window samples. Statistical features are extracted from each sample and a feature selection strategy is carried out using a genetic algorithm, and a forward selection and backwards elimination methodology. The four features derived from this process were used to construct an SVM classification model that detects presence of alcohol. The experiments yielded 7200 samples, 80% of which were used to train the model. The rest were used to evaluate the performance of the model, which obtained an area under the ROC curve of 0.98 and a sensitivity of 0.979. These results suggest that the proposed methodology can be used to detect the presence of alcohol and enforce prevention actions.

## 1. Introduction

One of the leading causes of death among young people are motor vehicle crashes [1], young drivers are 5 to 10 times more likely to experience injuries related to road crashes, and young males have a higher crash rate than young females [2]. There are several factors that may contribute to the increased number of crashes, such as social, situational, and exposure factors. Among social and situational factors include: the presence of passengers of similar age that may distract the driver [3], fatigue is also a risk factor among young people as they are affected by sleepiness more often [4], and social and economic status also plays and important role as they social group may affect their driving behaviors by encouraging them to take greater risks [5]. Recently, the grow of mobile phone usage have increased the risk of crashing among young people, due the increase in the level of cognitive and behavioral associated with people that use their phones while driving [6,7]. Alcohol consumption and drug usage while driving increases the crash risk for all drivers despite their age group affecting the cognitive process, thus increasing the risk of crashing [8,9].

On the other hand, exposure related factors include the weather condition, as it plays an important influence on the crash rates, as the young people exhibit less experience dealing with such conditions, such as snow, fog, rain, black ice conditions, etc., [10]. The type of road also affect the risk of crashing as urban, regional, and rural roads present different conditions [11]. The time also increase the risk for young people as their are more likely to crash at night and over the weekend [12], as we can see those risk factors plays an important role, thus measuring the safety efficiency of the drivers is very important [13].

Among all motor vehicle crashes involving young people, 25% of crash-related deaths among child passengers aged less than 15 years involves alcohol use [1]. Alcohol consumption while driving is illegal, nevertheless, and despite government penalties, the act of drinking and driving is a worldwide problem. In order to mitigate this issue, a wide variety of research has been conducted on smart systems able to detect this behavior. The research community has tried to develop smart systems that can be incorporated into next-generation vehicles in order to detect unsafely behaviors and prevent such accidents. These systems are known as infotainment systems, and their rapid development has turned traditional systems into smart-infotainment systems able to use contextual information to detect and react to changes inside the vehicle and inform the driver accordingly.

Recently, Wakana et al. [14] developed a portable device that uses a non-contact breath sensor to detect breath-based alcohol. The device measures the saturated water vapor in the human breath using gas sensors to detect ethanol; alcohol concentration was calculated using an algorithm based on a differential evolution method at each gas sensor’s output. The authors reported an accuracy of approximately ±10 ppm, however, the distance from the driver’s mouth to the sensor should be within 20 mm of distance. Another sensor-based approach was presented by Sandeep et al. [15], a novel Internet of Things (IoT) system that includes a touch sensor, an alcohol concentration sensor, facial recognition, heart rate measurement, and a GPS module. This systems aims at safeguarding drowsy drivers, but only the concept of the system was presented, neither an implementation nor a validation were conducted by the authors. Murata et al. presented a system capable of monitoring the condition of a driver by measuring biological signals using a custom seat with an air-pack sensor. Using a frequency time series analysis, the authors were able to determine whether drivers were intoxicated or not. Nevertheless, the authors reported that their system could not perform an accurate classification without baseline data of a non-drinking state for each subject [16].

Chen et al. [3] proposed a system to distinguish drunk driving from normal driving under simulated driving conditions, the author proposed a simulated system, using electromyogram, electrodermal activity, photo-plethysmography sensors, and a Tobii eye tracker, then, using a support vector machine, the systems detected normal and drunk driving; the authors reported an accuracy of 70%. On the other hand, Harkous et al. [17], presented a two stage machine learning method for drunk driving detection, the proposed methodology uses a series of sensors placed in the vehicle to feed a hidden Markov model that select the best subset of sensors to be used by a recurrent neural network, the system was based on the detection of the vehicle movement rather than the alcohol presence, the system achieved a 75–98% of accuracy depending of the number of sensors used by the model. Recently, Hyder et al. [18], developed a system based on an SoC (System on Chip), to detect drowsiness, the system uses a IoT sensor to detect the presence of alcohol, for this the system placed the alcohol detection sensor near the steering wheel to be close of the driver, then using a threshold, the presence of alcohol was detected, the system also detected the drowsiness by using cameras to detect the eye aspect ratio, the authors reported up to 92% of accuracy for the detection of the drowsiness when using the cameras, for the alcohol detection, only the threshold was reported. Vijayan et al. [19] also proposed a system to detect driver drowsiness based on the use of image processing, here the authors proposed a system that recorded the drivers’s face then was feed to deep neural networks to infer the state of the driver, the system used ResNet50, VGG16, and InceptionV3 to classify the driver’s state, the authors reported an accuracy of 76.16%, 71.22%, and 78.43%, respectively.

Another non-invasive approach was presented by Dai et al. [20]. They developed a system aimed at early detection and alert of dangerous vehicle maneuvers typically related to drunk driving. The system only needs information derived from the accelerometer and the orientation sensor of a mobile phone placed inside the vehicle; the system computes accelerations based on sensor readings, and compares them with typical drunk driving patterns extracted from real driving tests. The authors reported a false-positive rate of 0.49% and 2.39% in detecting abnormal curvilinear movements and speed control problems, respectively. Unfortunately, the performance of the system is heavily impacted by the phone placement and sliding. You et al. proposed a preliminary design for a personal alcohol tracking system with the aim to improve the reliability of current transdermal ethanol tracking devices to be used to raise the awareness of alcohol use, the system was able to detect the presence of alcohol using trans dermal alcohol concentration through the skin, nevertheless, the system showed a delay of 28–124 min from the ingestion up to detection [21].

An approach to road safety was also presented by Jamil et al., who proposed a system that uses a webcam coupled to a Raspberry Pi to detect blinks per minute as a measure of fatigue. The authors suggest that such a metric can be used to detect bad driving behaviors, although drinking was not tested. Additionally, their results demonstrated that different conditions, such as recently having a heavy meal, could affect the measuremente [22]; a similar approach was explored by Kulkarni et al. [23]. Finally, and related to the topic of ubiquitous approaches to detect unsafely behaviors, Celaya et al. [24] presented a system that detects when a subject is texting and driving. The authors used a wide angle camera inside the vehicle to record and analyze the behavior of the driver, detecting the use of a cellphone while driving by means of a deep neuronal network, with an accuracy of 0.89.

As it can be seen, most of the current approaches to detect drunk driving rely on external sensors that typically uses thresholds to detect such behaviors, such as comparing acceleration measurements with previously recorder unsafe patterns. Other researchers have developed systems that accurately detect alcohol, but such systems are heavily dependent on the sensors being close to the mouth of the driver or on having them wear a wristband with a transdermal sensor. We propose the use of low-cost IoT sensors to characterize the air and detect the presence of alcohol in the vehicle by processing the signals with genetic algorithms. In Section 2, the complete proposed methodology is detailed and the experimental setup is shown. Next, in Section 3, we show our findings. Finally, in Section 4 and Section 5, we comment on what these results mean, the main limitations of this work, and the next steps of this project.

## 2. Materials and Methods

**Figure 1:**
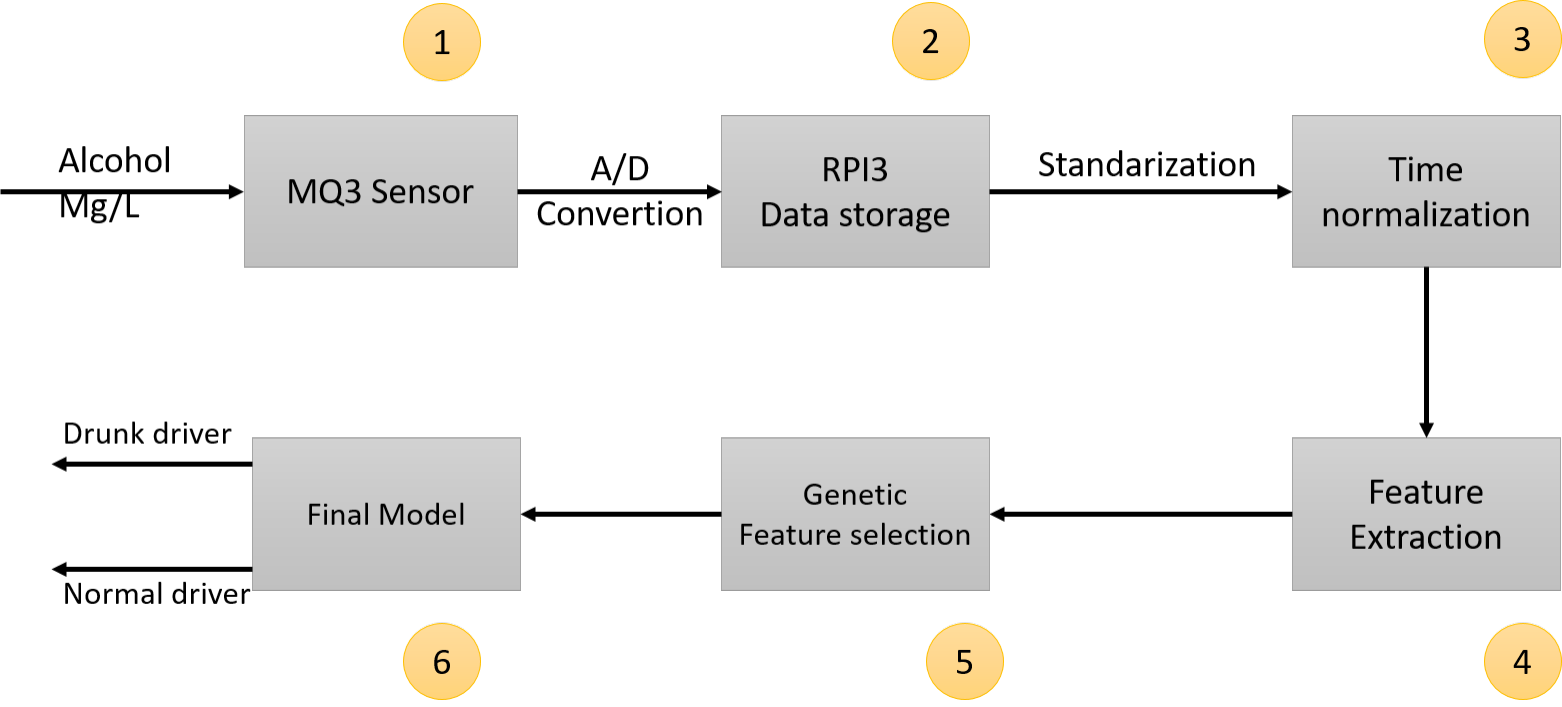
Flowchart of the proposed methodology.

A flowchart of the proposed methodology is presented in Figure 1. Briefly, in order to detect drunk drivers: (1) alcohol presence in the vehicle is measured and stored using seven alcohol sensors, (2) the measurements are standardized according to the sensor-specific sensibility and its longitudinal behavior, (3) statistical features are extracted from the normalized signals, (4) a genetic algorithm is used to train several models in order to find the optimal subset of features within the dataset, and (5) a model that accurately classifies drunk and non-drunk drivers is constructed. Each stage is further detailed in the following subsections.

### 2.1. Data Acquisition

**Figure 2:**
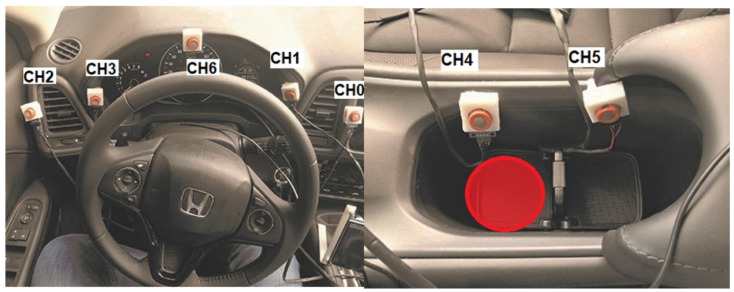
Layout of the sensor placement.

A total of seven low-cost MQ3 sensors were placed near the driver in a test vehicle, a Honda HR-V 2018 with all windows closed. The locations of the sensors were chosen so that they would not hinder drivability, but were close to either air flow from the air conditioning vents and/or to drink holders. Figure 2 shows the exact place where each sensor was installed. The MQ3 sensors are low-cost metal oxide semiconductor devices that can detect the presence of alcohol vapor at concentrations ranging from 0.05 mg/L to 10 mg/L. This sensors work by measuring conductivity; the higher the higher the concentration of alcohol vapor, the higher the conductivity. This device has a sensing element made of Aluminum Oxide (AL${}_{2}$O${}_{3}$) based ceramic and has a coating of Tin Dioxide (SnO${}_{2}$), the Tin Dioxide is sensitive towards alcohol, thus, the ceramic heats the Tin Dioxide and forms a sensor by changing the resistance when the particles of Oxygen are absorbed by the SnO${}_{2}$ surface, in the presence of alcohol, however, the surface density of adsorbed oxygen decreases as it reacts with the alcohols, which lowers the resistance and changing the generated value by the sensor. The sensor has a high sensitivity to alcohol and a low sensitivity to smoke and gasoline [25].

For our experiment, samples were obtained from four 30 min sessions, two with no alcohol in the vehicle and two with a 10 mL sample of $90^{\circ }$ ethanol alcohol in a drink holder between seats (red circle in Figure 2), a typical place for drivers to place their beverages when driving, to avoid any external factor that may increase the levels when present; all the people inside the vehicle had not drank any alcohol. This was implemented as the goal of this research is to first detect the presence or absence of alcohol inside the vehicle, and then in a future research, infer who is drinking/drunk. Experiments were carried out on different days to lower the risk of cross-contamination. Data were acquired with a sample rate of 2 Hz, yielding a total of 14,400 samples. MQ3 sensors produce analog signals; we carried out an analog-to-digital conversion with a 10-bit MCP3008 converter and a custom board.

### 2.2. Data Standardization

**Figure 3:**
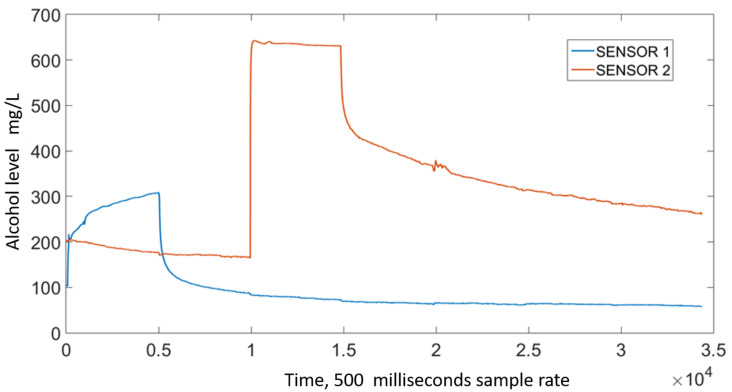
Output from two MQ3 sensors exposed to the same alcohol sample.

**Figure 4:**
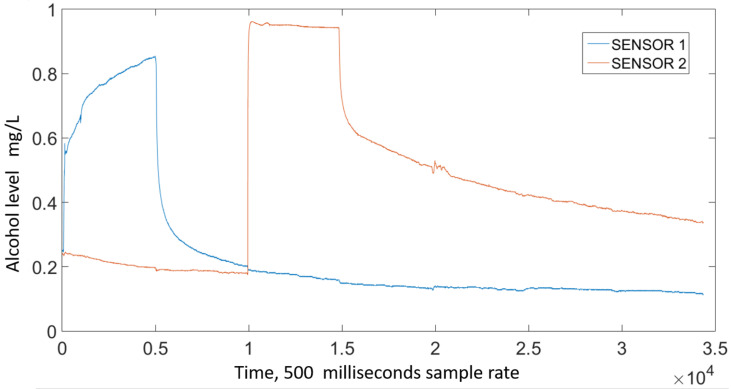
Standardized signal from two MQ3 sensors exposed to the same alcohol sample.

The signal from the MQ3 sensors exhibit a sensor- and time-dependent variation in amplitude. Regarding the former, Figure 3 shows the output signal for two sensors reacting to the same sample, where a noticeable difference in amplitude can be seen. To account for this variation, a sensor-specific standardization was carried out; new values were defined using Equation (1), where *Y* represents the standardized value, *V* the output from a specific sensor, and $V_{m}in$ and $V_{m}ax$ the minimum and maximum amplitudes measured using that specific sensor. The standardized signals exhibit a 0–1 range, Figure 4 shows the signals from Figure 3 after this standardization took place.

(1) $$Y=\frac{V-V_{min}}{V_{max}-V_{min}}.$$

**Figure 5:**
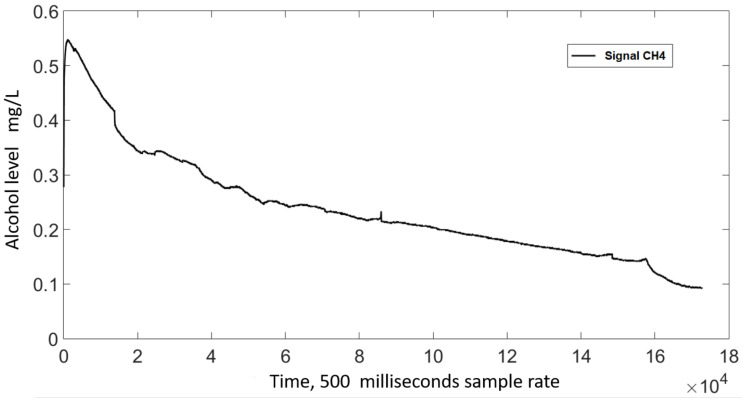
Longitudinal behavior of a sensor without an alcohol sample.

We also noticed that the signal from the sensors was time-dependant regardless of whether alcohol was present or not; the sensor decreased their output as time went by. Figure 5 shows the standardized signal value obtained from a sensor during a 24 h experiment with no alcohol nearby, as it can be seen, the standardized signal goes from 0.55 to 0.1.

**Figure 6:**
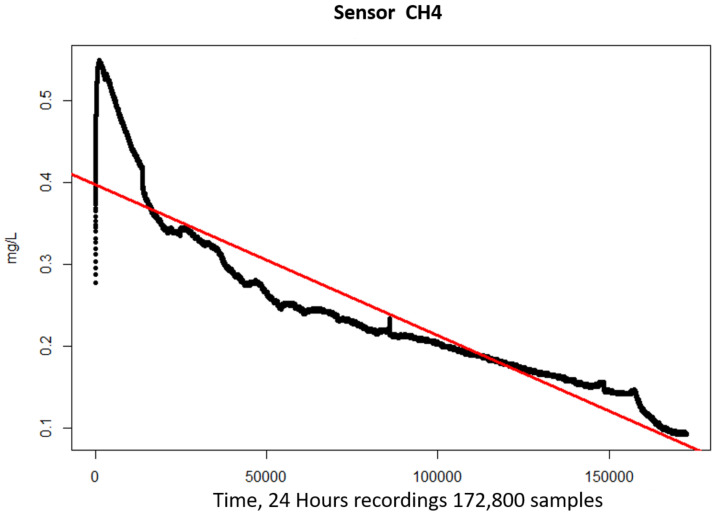
Fitted line obtained after performing a linear regression.

In order to remove this time dependency, a linear regression (Equation (2)) was used to adjust the value of the signal for each sensor. Here, $X_{i}$ is the *i*th standardized value for each sensor, $Y_{i}$ its corresponding regression value, and $\beta_{0}$ and $\beta_{1}$ represent the offset and slope of the fitted line, respectively. Figure 6 shows, in red, the fitted line yielded from the regression analysis performed to the sample from Figure 5.

(2) $$Y_{i}=\beta_{0}+\beta_{1}\cdot X_{i}$$

The linear regression was applied to sensors CH0–CH6 obtaining an $R^{2}$ of 0.8091, 0.8540, 0.8541, 0.8902, 0.8613, 0.8396, and 0.7518, respectively. These values indicate a very good fit for each sensor, thus allowing for the removal of the time dependency in the data.

**Figure 7:**
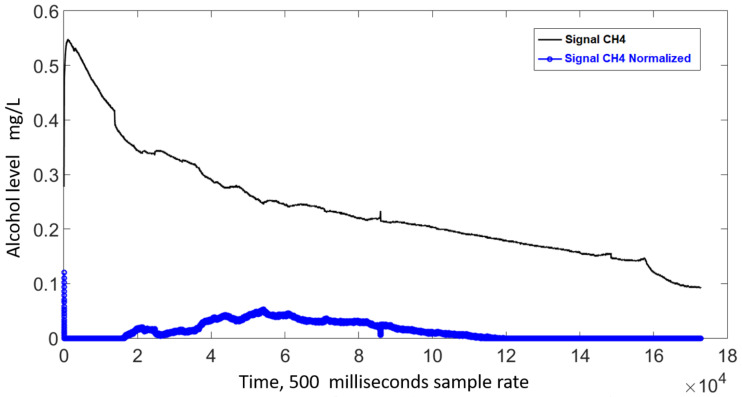
Time-adjusted signal.

Finally, the fitted line is subtracted from the standardized signal in order to remove the time dependency. Figure 7 shows, in blue, the time-adjusted signal, first 10 s of the standarized data set were omitted to remove the outliers generated by the initial heat up of the sensor shown on Figure 7.

### 2.3. Feature Extraction

Recently, the community has presented novel approaches to perform alcohol detection inside the vehicle; nevertheless, many of the approaches rely only on simple thresholds or a combination of digital filters mainly dealing with the suppression of the noise. However, our approach takes advantage of machine learning algorithms [26,27]. In order to characterize the signal accurately and perform detection in quasi real-time conditions, data were split into five-second windows. Eight features, detailed in Table 1, were extracted from each window, yielding a database with 1440 observations and 56 features. The selection of features to extract was based on previous work with time-dependent signals where we tackle a similar problem, trying to characterize a signal coming from an analog sensor; our work demonstrated that the first statistical moments (1–4) along with the Max and dynamic range features could successfully characterize a time dependent signal and achieve a highly accurate model [28].

### 2.4. Feature Selection

The dimension of our database was presented in the previous section. Finding an optimal model within a 1440 × 56 matrix usually becomes a computational challenge, therefore, we propose a feature selection process guided by a nature-driven approach. This kind of processes has recently gathered attention because of the lower computational requirements needed to solve complex problems [29]. From these, some of the most powerful methods are evolution-driven approaches, such methodologies take advantage of the evolutionary process presented by Charles Darwin [30]—the processes generate models with features that reproduce, mutate, and migrate, following the evolutionary theory where the fittest models prevail generation after generation leaving low-fittest features behind. For this research, we used a novel library of genetic algorithms called GALGO [31], a powerful multivariate feature selection based on genetic algorithms.

The genetic algorithm evolved a set of random multivariate models following the evolution theory, thus generating highly accurate models [28]. The highest the frequency in which a feature appears in these models, the greater its importance in detecting drunk drivers; therefore, the frequency of the features was used to sort and rank them. Then, a forward selection and backwards elimination process was used to select the best performance model keeping the number of features low [28].

The data set used for this research was split into two sets: the first “training” with (80%) of the samples and the “test” set with (20%) of the samples. The genetic search was then implemented to search for the best performing model using only the training data set, keeping the model construction isolated from the test set. One thousand random five-feature models were evolved throughout 200 generations; while performing this search, fitness was evaluated as the accuracy of each model following a 3-fold cross-validation strategy (using 70% to train the model and 30% to validate the model). This was performed using the previously defined train samples; the test set was not used at this stage, genetic parameters were chosen as suggested by Treviño et al. [31]. A support vector machine (SVM) function with a radial kernel was used as the classifier. The SVM function maps the training examples to points in space so as to maximize the width of the gap between the two categories, new instances are then mapped into that same space and predicted to belong to a category based on which side of the hyper-plane they belong [31,32].

**Figure 8:**
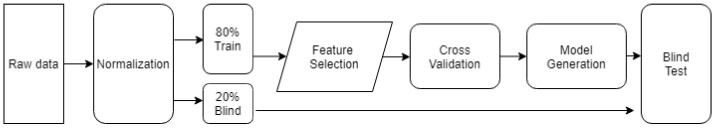
Model generation and validation methodology.

After performing the genetic search using GALGO, the forward selection and backwards elimination processes were computed using the whole training data set (80% of the whole data). For this, the forward selection algorithm used the ranking generated by GALGO to construct models adding one feature at a time, in this stage, when adding a feature to the model, the accuracy was checked, if the model + the new feature achieved a high accuracy, the feature was kept, and the rest were disregarded. Then, the backward elimination process was performed to avoid redundant information and further reduce the amount of features to be used. This process evaluated the model generated by the forward selection strategy, then the process removed one of the features to check the accuracy. If the model had not decreased its performance, that feature was removed from the final model, otherwise, the feature was kept. This process was repeated until all features were checked to vary its impact on the accuracy of the model. In order to avoid overfitting, a cross-validation was performed in the training stage, here, using the features found by the genetic process, a 5-fold cross-validation was performed to train and test the model and assess bias towards a specific data partition. In Figure 8, the detailed process for the model generation is presented.

Once the final model was constructed following said strategy, in order to measure the true performance, the model was evaluated using the test subset (20% of the kept unseen samples).

## 3. Results

**Figure 9:**
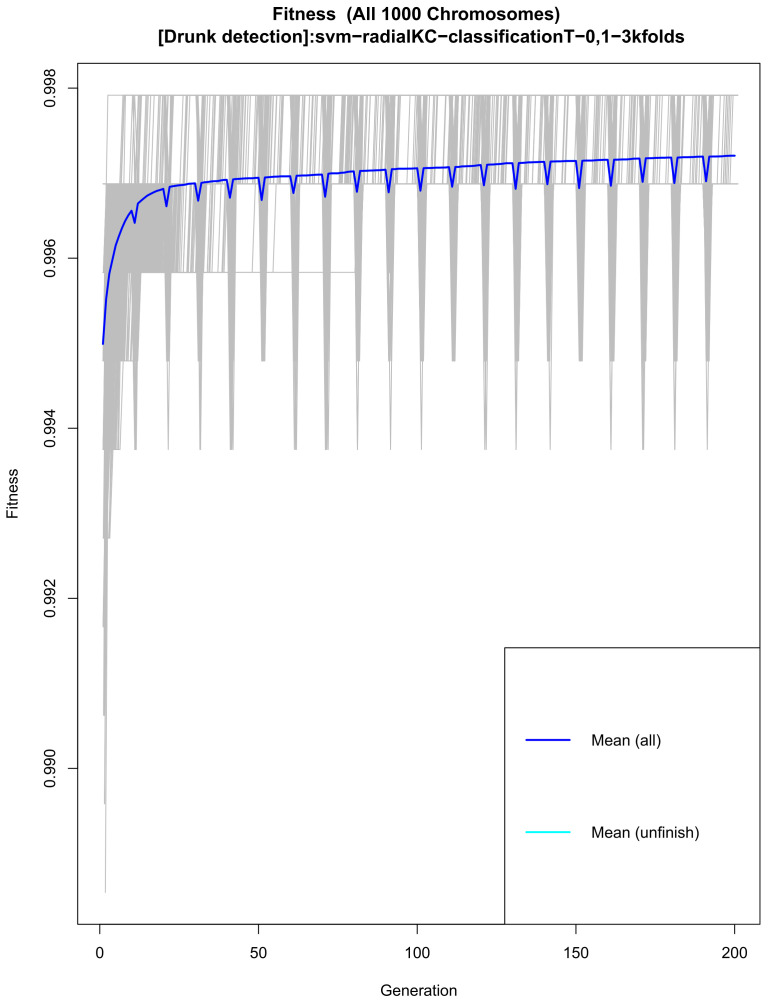
Average fitness of the models throughout the 200 generations.

**Figure 10:**
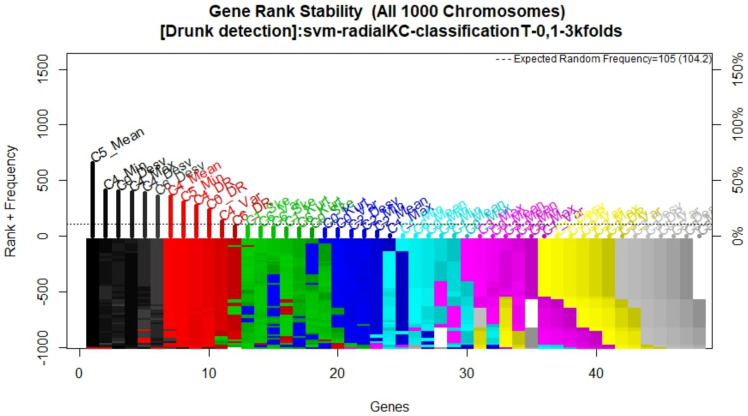
Feature rank stability.

Data collection resulted in a total of 1440 5 s recordings, with 56 features and a ground true label extracted from each, yielding a 1440 × 57 matrix. Using this data set, the genetic search generated 1000 models that evolved over 200 generations each. Figure 9 shows the average model accuracy as models evolved and highlights that accuracy had converged, that is, no more generations were needed. Similarly, Figure 10 shows that the frequency in which features appeared in the models had stabilized. There, it can be seen that the eleven most frequent features are above the expected random frequency; thus, even with more models or more generations, the rank of the most frequent features would not have changed.

**Figure 11:**
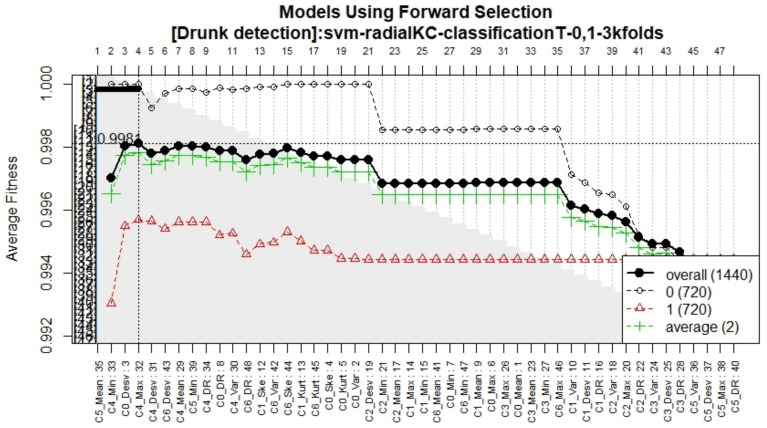
Accuracy of the models during the forward selection methodology.

**Figure 12:**
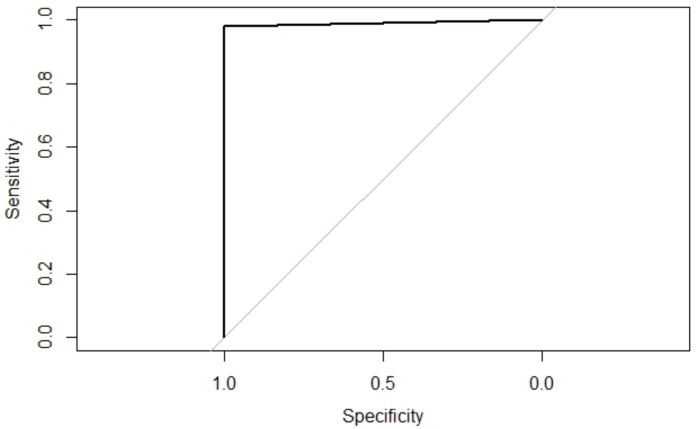
ROC curve of the model on test data set.

As shown in Figure 11, the forward selection process yielded a 4-feature model. Since no features were discarded with the backwards elimination process, the final model was constructed with only four features, detailed in Table 2. Using these four features, a support vector machine model was trained using the 80% training data set. To measure its performance, the area under the Receiver Operator Characteristic (ROC) Curve (AUC) was computed. Figure 12 shows the ROC curve for the training data set; the model achieved an AUC of 0.9800 and an accuracy of 0.9800 with a 95% Confidence Interval (CI) ranging between 0.9720 and 0.9880.

In order to test for a specific bias towards the data partition, a k = 5 cross-validation strategy was performed using the 80% training data set; details are presented in Table 3. Finally, to test the real performance on unseen data, the model was also evaluated using the 20% unseen test data set, yielding an AUC of 0.9896 and an accuracy of 0.9896 with a 95% CI of 0.9779–1.0.

**Figure 13:**
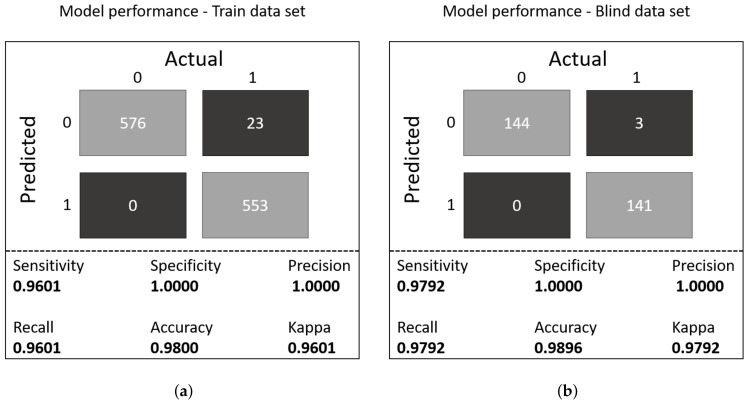
Model performance on train/test samples: (**a**) confusion matrix for the train samples. (**b**) confusion matrix for the test samples.

Figure 13 shows the confusion matrix for the model evaluated using the train and test sets. For the train data set, the model achieved a sensitivity of 0.9601 with an specificity of 1.0; for the test data set, the model achieved a sensitivity of 0.9792 with a specificity of 1.0; the model misclassified only three observations.

## 4. Discussion

The proposed methodology was able to demonstrate the effectiveness of using genetic algorithms and machine learning systems in smart cars; our model was able to detect the presence of alcohol, inferring it would also be able to detect a drinking and driving situation. The methodology includes the collection of raw data from the MQ3 sensors, standardizing and time-adjusting the data, transforming them into five-second windows, extracting statistical features, selecting the most relevant ones using genetic algorithms, and training and evaluating the final model. The standardization stage removed the variability in sensibility between sensors, and the linear regression eliminated longitudinal variability, allowing for these sensors to be used in vehicle applications. Using the statistical features, 1000 random models were evolved throughout 200 generations using a 3-fold cross-validation, producing a feature ranking based on their frequency, thus ensuring that only the most relevant features would be included in the final model. A forward selection and backwards elimination process was included to make the final model as accurate and small as possible. Finally, the performance of the model was evaluated using the test data set, i.e., samples that had not been used previously to train the model or during the feature selection process.

The final model included the following features: average amplitude of the signal from channel 5, minimum value of the signal from channel 4, standard deviation of the signal obtained from channel 0, and maximum value of the signal from channel 4. It is important to notice that, while many approaches use only a signal value over a threshold in order to detect the presence of alcohol, the proposed model also includes other information, such as the standard deviation of the signal. Additionally, the fact that none of these features were discarded under the backwards elimination process also suggests that all features contribute significantly to the performance of the multivariate model. Further, it is also relevant that the model is constructed with information from sensors placed in different locations, suggesting that sensing in different areas at the same time is needed to perform this task accurately. Furthermore, the sensors whose information is included in the final model are the three sensors closest to the sample; further experiments where the sample is located near the door of the driver should help to identify whether the positions of the other four sensors are adequate. The performance of the final model was evaluated in two ways, first using a cross-validation approach with the training data, and then performing a blind test with the test samples. In both cases, the model achieved AUC values just shy of 1.000, suggesting that the model is robust and therefore would perform accurately with new samples.

We propose the use of a multivariate model for alcohol detection targeting quasi real-time applications to detect drinking and driving behaviors. The presented methodology builds an SVM classifier with four features derived from a genetic algorithm approach; the procedure is accurate and robust. Once the model is trained and refined, the model could be transferred to a smart infotainment system to detect unsafely behaviors. The proposed methodology uses a 5 s window to analyze changes in the air and detect the presence of alcohol. Comparing this methodology with other approaches, there is no need to exhale near the sensor as with the system proposed by Wakana et al. proposed [14], and the speed of the detection is quasi real-time, unlike that presented by You et al. [21], which takes between 24 and 124 min.

One key component of the proposed methodology is the incorporation of the genetic algorithms to perform the feature selection process, to validate the performance of such an important process, we compared our approach (GALGO + SVM) to a commonly used step-wise feature selection algorithm (LASSO) [33,34], to asses the performance of the genetic selection vs step-wise selection we used the exactly same data sets (80% train, 20% test) to search for the best subset of features to construct a representative model, afterwards, the same metrics were computed for train and test. LASSO methodology yielded in a representative model with 14 features, Table 4 shows the breakdown of the selected features.

Then we constructed two SVM models, one with a radial kernel as our approach, then a linear kernel due the linearly optimization performed by LASSO, with those models we compared the LASSO performance to our results using the GALGO + SVM model with a radial kernel and a new GALGO + SVM with a linear kernel in order to compare it with the LASSO model. Table 5 shows the performance of LASSO compared to our approach, as we can see, the LASSO + SVM (linear) performs very similar to the presented genetic search; nevertheless, the LASSO model contains 14 features compared to only four features selected by our approach—this increase in the number of features tends to exhibit a bias towards the data set, this is shown in the LASSO + SVM (Radial) row, where the performance is lower with sensitivity of 0.559 and 0.4861 for the train and test sets, respectively. Nevertheless, our approach with only four features exhibit almost the same performance despite the kernel is being used, demonstrating that the features selected by the genetic search are very robust.

This work has some limitations, the most important one being that we only analyzed the alcohol sample in one location. Further, all samples were derived from the same vehicle; however, the promising results lead us to think that even if not the model, at least the methodology will easily adapt to other vehicles and other placements of the alcohol source. That is, once the model is trained with more diverse data, the model will only become more robust. Another limitation was that the experiments were performed with the windows closed; opening the windows may have a significant impact in the amplitude of the signals from the sensors. Another possible limitation is the presence of other people that have consumed alcohol inside the vehicle, as the concentration of Alcohol/O${}_{2}$/CO${}_{2}$ will vary, thus this behavior could impact in the alcohol detection rate, the size of the vehicle along with the ventilation systems will play a role in the detection rate as each vehicle will circulate the air in a different way, to solve this problem each type of car should train a specific model to search for the best combination of sensors, to allow the correct identification of alcohol inside. Nowadays, more vehicles are being equipped with high efficiency filtration systems such as HEPA filters, for those filtration systems; the proposed methodology should sense the air before passing through the filtration system in order to minimize the impact on the detection rate. Nevertheless, having seven sensors in the vehicle would allow for at least a few of them to be close to the source of alcohol, lowering the impact of having the windows open; however, this is a variable that will have to be further analyzed in the future.

## 5. Conclusions

We collected raw data from seven MQ3 sensor placed in strategic but not intrusive places inside a vehicle. Data were standardized, time-adjusted, and transformed into 5 s windows. Eight statistical features were derived from each 5 s window, yielding a 1440 samples × 56 features database that was and split into train and test sets with a 80–20 ratio. Features were ranked using the train set and a genetic algorithm, and using a forward selection and backwards elimination procedure, four features were selected. Still using the train set, a model was trained and validated with a 3-fold cross-validation strategy. Finally, the performance of the model was assessed using the test set, yielding an accuracy of 0.98 and an AUC of 0.989. The model successfully detected the presence of alcohol inside the vehicle in quasi real-time, thus detecting a potential drinking and driving behavior. The system takes into account features that were not previously studied by other authors as well as information from three different locations, indicating that such places may be optimal sensor locations. This system could be used to alert someone once alcohol is detected, avoiding potential accidents.

## 6. Future Work

For the future of this research we propose the analysis of different concentrations of alcohol to assess the sensitivity of the proposed methodology. The influence of factors such as the influence of the type of vehicle, and the ventilation systems will be the subjects of further study. External factors such as the impact of people that have consumed alcohol will be explored to assess the detection rate in such scenarios. To further improve the methodology, the incorporation of image-based recognition modules to monitor the driver and passengers, merging both modules and developing a meta-heuristic system will be explored. Then, the systems will be capable of reacting based on the alcohol in the vehicle and also the behavior of the driver and passengers.

## Figures and Tables

**Table 1:** Extracted features.

Feature	Formula
Mean (M1)	$\bar{x}=\frac{1}{n}\sum_{i=1}^{n}X_{i}$
Variance (M2)	$\sigma^{2}=\frac{\sum_{i=1}^{n}{\left(X_{i}-\bar{X}\right)}^{2}}{N}$
Skewness (M3)	$\gamma_{1}=\frac{\frac{1}{n}\sum_{i=1}^{n}{\left(x_{i}-\bar{x}\right)}^{3}}{{\left[\frac{1}{n-1}\sum_{i=1}^{n}{\left(x_{i}-\bar{x}\right)}^{2}\right]}^{3/2}}$
kurtosis (M4)	$K=\frac{\sum_{i=1}^{N}{\left(X_{i}-\bar{X}\right)}^{4}}{N\sigma^{4}}-3$
Standard Deviation	$\sigma =\sqrt{\sigma^{2}}$
Max	$X_{\left(max\right)}=\max\left\{\;X_{1},\ldots ,X_{n}\;\right\}$
Min	$X_{\left(min\right)}=\min\left\{\;X_{1},\ldots ,X_{n}\;\right\}$
Dynamic range	$DR=X_{\left(max\right)}-X_{\left(min\right)}$

$X_{i}$ is the *i*th value within the 5 s window being processed.

**Table 2:** Model features.

Feature	Sensor Channel
Mean	5
Min	4
Std. Deviation	0
Max	4

**Table 3:** Cross-validation performance of the proposed methodology.

	Train				Test			
***i***	**AUC**	**Accuracy**	**Sensitivity**	**Specificity**	**AUC**	**Accuracy**	**Sensitivity**	**Specificity**
*1*	0.9555	0.9555	0.9111	1	0.9565	0.9565	0.913	1
*2*	0.9663	0.9663	0.9326	1	0.9784	0.9784	0.9569	1
*3*	0.9751	0.9751	0.9501	1	0.9826	0.9826	0.9652	1
*4*	0.9957	0.9957	0.9913	1	0.9913	0.9913	0.9826	1
*5*	0.9772	0.9772	0.9544	1	0.9739	0.9739	0.9478	1
**Average=**	**0.97396**	**0.97396**	**0.9479**	**1**	**0.97654**	**0.97654**	**0.9531**	**1**

**Table 4:** LASSO Model features.

Feature	Sensor Channel
Skewness	0, 1
Kurtosis	0, 1
Mean	3, 1
Min	0, 1, 3
Dinamic range	4
Max	0, 2, 4, 5

**Table 5:** Genetic feature selection (GALGO) vs, step-wise feature selection (LASSO).

		Train			Test		
**Strategy**	**# of Features**	**AUC**	**Sensitivity**	**Specificity**	**AUC**	**Sensitivity**	**Specificity**
***GALGO + SVM (Radial)***	***4***	***0.98***	***0.9601***	***1***	***0.9896***	***0.9792***	***1***
GALGO + SVM (Linear)	4	0.9759	0.9565	1	0.9826	0.9652	1
LASSO + SVM (Radial)	14	0.7214	0.559	0.8837	0.6944	0.4861	0.9028
LASSO + SVM (Linear)	14	0.9948	0.9896	1	0.9965	0.9931	1

## Data Availability

Not applicable.
